# Self-reported race/ethnicity in the age of genomic research: its potential impact on understanding health disparities

**DOI:** 10.1186/s40246-014-0023-x

**Published:** 2015-01-07

**Authors:** Tesfaye B Mersha, Tilahun Abebe

**Affiliations:** Division of Asthma Research, Department of Pediatrics, Cincinnati Children’s Hospital Medical Center, University of Cincinnati, Cincinnati, OH USA; Department of Biology, University of Northern Iowa, Cedar Falls, IA USA

**Keywords:** Genome, Race, Ethnicity, Ancestry, Ancestry informative markers, Ancestry haplotype, Admixture, Health disparity

## Abstract

This review explores the limitations of self-reported race, ethnicity, and genetic ancestry in biomedical research. Various terminologies are used to classify human differences in genomic research including race, ethnicity, and ancestry. Although race and ethnicity are related, race refers to a person’s physical appearance, such as skin color and eye color. Ethnicity, on the other hand, refers to communality in cultural heritage, language, social practice, traditions, and geopolitical factors. Genetic ancestry inferred using ancestry informative markers (AIMs) is based on genetic/genomic data. Phenotype-based race/ethnicity information and data computed using AIMs often disagree. For example, self-reporting African Americans can have drastically different levels of African or European ancestry. Genetic analysis of individual ancestry shows that some self-identified African Americans have up to 99% of European ancestry, whereas some self-identified European Americans have substantial admixture from African ancestry. Similarly, African ancestry in the Latino population varies between 3% in Mexican Americans to 16% in Puerto Ricans. The implication of this is that, in African American or Latino populations, self-reported ancestry may not be as accurate as direct assessment of individual genomic information in predicting treatment outcomes. To better understand human genetic variation in the context of health disparities, we suggest using “ancestry” (or biogeographical ancestry) to describe actual genetic variation, “race” to describe health disparity in societies characterized by racial categories, and “ethnicity” to describe traditions, lifestyle, diet, and values. We also suggest using ancestry informative markers for precise characterization of individuals’ biological ancestry. Understanding the sources of human genetic variation and the causes of health disparities could lead to interventions that would improve the health of all individuals.

## Genetic variation in the human genome

The human genome is composed of over three billion bases of DNA and contains between 25,000 and 30,000 protein-coding genes [1]. On average, any two randomly selected humans have 99.9% identical DNA [2]. Yet, these 0.1% differences spreading over the entire genome contribute to genetic heterogeneity that uniquely distinguishes each person. Because the majority of the human genome contains non-coding DNA, the bulk of this genetic diversity is not visible at the phenotype level. Variable regions on the genome are broadly classified into single nucleotide polymorphisms (SNPs) and structural variations (SVs). SNPs are changes in single DNA bases whereas SVs involve large genomic changes including indels and genomic rearrangements (translocation, transversion). The *International HapMap Project* was the first multi-institutional effort to catalog variations and develop a haplotype map (HapMap) of the human genome. The HapMap project had identified over 5 million SNPs in the human genome including their distribution among people in different parts of the world [3]. While successful, the HapMap project had two major limitations: 1) it encompassed only SNPs, and 2) it only contained the most common genetic variants (those with frequencies >5%). Many genetic disorders are caused by rare SNPs (with frequencies <5%) and by SVs. The 1000 Genomes Project was formed in 2008 to sequence and generate a catalog of human genetic variation and haplotypes from the genomes of at least 1,000 people around the world (hence the name the 1000 Genome Project). The current phase 3 analysis of the project contains 2,535 individuals from 26 populations and identified a total of over 81 million variants, ranging from SNPs, indels, and other small variants to insertions of mobile elements and large structural variants spanning 100 s of kilobases (http://www.1000genomes.org/). This haplotype resource at finer scales will facilitate the understanding of genetic variation at genomic and geographic levels [4].

Because of their sheer number, SNPs are the major sources of genetic and phenotypic diversity, accounting for 95% of all known sequence variations [5]. Different versions of the DNA bases present at a SNP locus are referred to as *alleles*. Alleles with a frequency greater than 5% are called common variants, those with a frequency of 1%–5% are low frequent variants and those less than 1% are rare variants. Because rare variants might have arisen after populations diverged or occurred in recent human history, they are more likely to be population-specific and, therefore, they may not be shared with different populations. Thus, the overrepresentation of rare causal variants in certain population could explain the observed differences in disease prevalence, including asthma [6].

There are two potential reasons why some variants are relatively common in one population but absent (or nearly so) in another: a) a recent emergence of a variant that has not yet had time to spread to other populations and b) natural selection in a specific local environment. An example of the first scenario is a SNP that causes hereditary hemochromatosis, which is common in Europe but very rare elsewhere. Lactase persistence is an excellent example of the influence of natural selection on allelic frequency. Lactase persistence into adulthood is prevalent in Somali camel herders from Ethiopia where milk consumption continues beyond childhood [7]. Positive selection in a geographic-specific manner has also been seen in genes that affect skin pigmentation [8] and resistance to malaria [9].

## Human ancestry

Anatomically, modern humans first appeared in Africa some 150,000 to 200,000 years ago [10]. About 60,000 years ago, humans left Africa in waves of migrations and, through a sequential chain of colonies, spread to occupy most of today’s land masses. During this journey, they encountered different environments and climates and came in contact with novel pathogens and animals. They formed local communities, separated by geographic, linguistic, cultural, and social barriers. Mutation, genetic drift, and natural selection operated in parallel with demographic and historical events to weave the patterns of human variation in extant populations. The result of this interplay was the imprint of genetic ancestry and population structure carried in the genome of each individual and groups that lead to the development of the remarkable racial and ethnic diversity that we see today.

**Figure 1:**
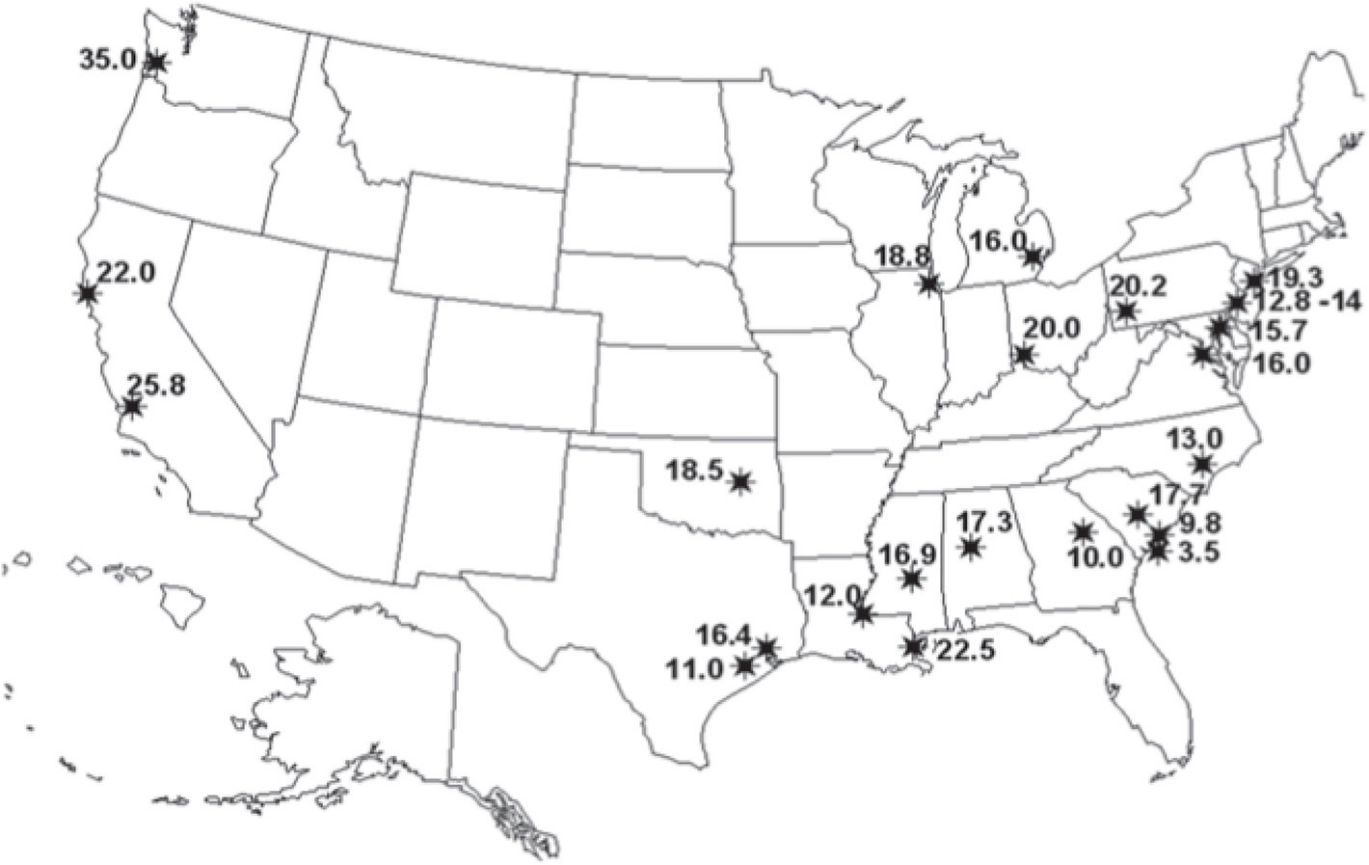
**Map showing estimates of the percentage of European contribution to several African American communities throughout the US.** The percentage of European contribution to several African American samples within the continental US varies tenfold, from 3.5% in the isolated Gullah-speaking Sea Islanders from South Carolina to 35% in Seattle. Reproduced from Parra [15].

**Figure 2:**
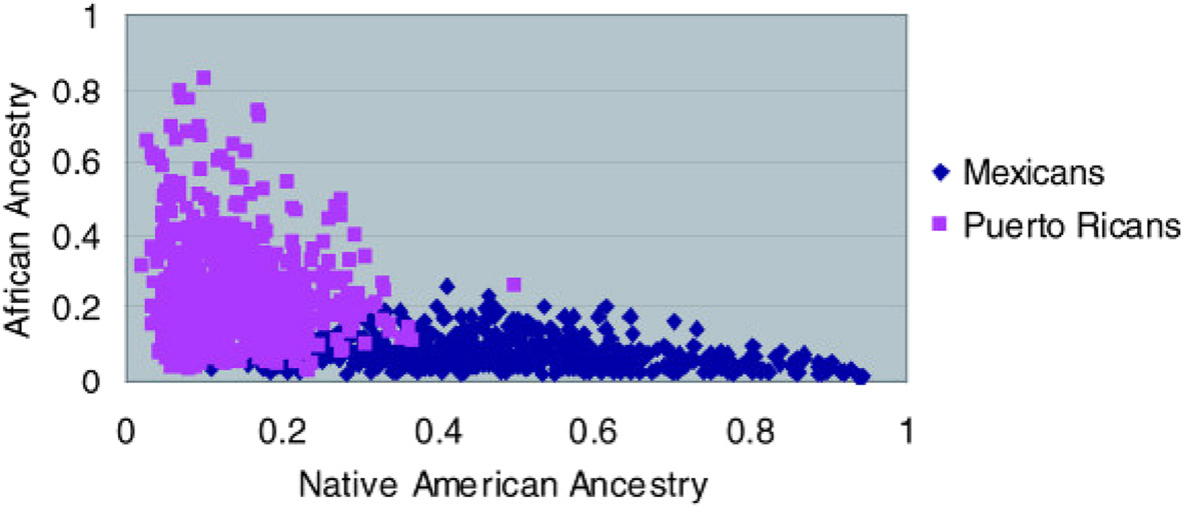
**Ancestry proportions of Mexicans vs. Puerto Ricans.** Although Mexicans and Puerto Ricans are both considered Latino or Hispanics, Mexicans, on average, have a higher proportion of Native American ancestry (35%–64%) but a lower proportion of African ancestry (3%–5%). Puerto Ricans have lower proportion of Native American ancestry (12%–15%) and higher proportion of African ancestry (18%–25%). Reproduced from Risch et al. [22].

Race and ethnicity are widely used interchangeably in population research and incorporate cultural, linguistic, biological, and geopolitical factors [11]. Although its use is primarily social, the term “race” is commonly defined in the scientific literature to refer to biological differences (such as skin color) between groups assumed to have different biogeographical ancestries or genetic makeup [11]. It is a “construct of human variability based on perceived differences in biology, physical appearance, and behavior” [12]. To the contrary, ethnicity is a complex multidimensional construct that reflects biological factors, geographical origins, historical influences, as well as shared customs, beliefs, and traditions among populations that may or may not have a common genetic origin [13]. For example, the Caucasian race contains such ethnicities as German, Irish, Spanish, and French each with their own culture, language, and tradition. Self-reported race/ethnicity is frequently used in epidemiological studies to assess an individual’s background origin. Often times, participants in the US are asked to specify a single race/ethnic group based on six categories: White, Black, Black Hispanic, White Hispanic, Asian, or other. Most questionnaires do not offer an opportunity for participants to choose multiple responses on their ancestral heritage. Most often, one family member declares for the rest, thus preventing detailed analysis of individuals with multiple (and differing) origins. A child of mixed parents (one black and one white) is socially classified as black, even though genetically, the child could just as easily be considered white (genotype 50/50). This classification was based on historical mandate of the “one-drop rule,” which stated that any individual with African ancestry would be considered a member of the Black race [14]. African and European ancestry in self-identified African Americans can vary wildly with proportions of European ancestry spanning the full range of variation, which can have significant impact on how we identify disease loci using genetics approach [13]. Parra [15] presents data showing that the percentage of European contribution to several African American communities within the continental US varies tenfold, from 3.5% in the isolated Gullah-speaking Sea Islanders from South Carolina to 35% in Seattle (Figure 1). Another example with broad ranges variation in admixture is the “Hispanic” or “Latino” population. The use of a single Hispanic or Latino ethnic category is insufficient for characterizing genetic background associated with Hispanics or Latinos because Hispanics have variable proportions of European, Native American, and African ancestry [16], as well as disease prevalence including asthma [17]. Mexican Americans, on average, have a higher proportion of Native American ancestry (ranging from 35% to 64%) but a lower proportion of African ancestry (ranging from 3% to 5%) than Puerto Ricans (Native American ancestry ranges between 12% and 15% and African ancestry ranges between 18% and 25%) [18-20] (Figure 2). Such higher proportion of African ancestry in Puerto Ricans could be the reason why the prevalence of asthma is the highest among Puerto Ricans (19.9%) and the lowest among Mexican Americans (6.5%). This phenomenon is referred to as the “Hispanic Paradox” [21].

Although on average, populations that are geographically close to one another show stronger correlation and higher genetic similarity than geographically separated populations and substantial differences in allele frequencies are also observed within geographic regions [23]. Several studies including ours showed that genetic diversity in humans is higher between individuals of the same race (~85%) than between races (~15%) [4,24]. A good example is the wide variation observed in two African populations. The prevalence of HLA-B*5701 variant in the Masai group in Kenya is 13.6%; the frequency of the same allele was zero among the Yoruba in Nigeria and 5.8% among European ancestry. Another seminal study is the complete sequence of two US scientists of European origin, namely, James Watson and Craig Venter, and an Asian scientist, Seong-Jin Kim. The two Europeans share fewer SNPs (461,000) than they each share with Seong-Jin Kim (569,000 and 481,000, respectively) [25-27]. On the basis of the subjects’ physical appearance, one would consider Venter’s DNA, and not Kim’s, a better approximation of Watson’s DNA. These results reflect a well-known feature of human diversity, that is, different genetic polymorphisms are distributed over the world in a discordant manner [28,29]. These observations reveal characterization of races simply as “White” or “Caucasian”, “Asian”, “African”, or “Latino” which are poor predictors of human biological diversity or similarity. Thus, although race/ethnicity categories are helpful to study socio-cultural and traditional values within groups and can help cluster individuals coming from geographically distant regions, they do not reveal the extent of admixture in an individual with admixed ancestry (Table 1). This is because an admixed individual can have multiple ancestries through intermixing (e.g., ‘Latino’) [30]. Group identity (for example, Hispanic American vs. African American) and genetic heritage are much more complex than self-identity. Although skin colors are often associated with race, individuals with light skin or dark skin could have an appreciable number of black or white ancestry genes, respectively. This is because visual classification of skin color is interpreted differently by patients, health care workers, and family doctors [31,32]. For example, studies in Cuba showed that the same individual can be classified into different color categories: family doctors tend to classify them as darker, while health care workers tend to classify them as lighter [31,32]. In addition, two people with the same level of pigmentation (melanin index) and skin color in two different parts of Cuba could be classified into different color categories. In Villa Clara Province, a person would be identified as mestizo, while in Santiago de Cuba, where more of the population is darker-skinned, a person with the same pigmentation could be classified as white [33]. Using autosomal ancestry markers, 72% of Cuban genes have European descent, 20% African, and 8% Native American [33,34]. Similarly, in Brazil, the correlation between biogeographic ancestry and categories of skin colors are region-dependent, relatively stronger correlations in Salvador (*r* = 0.585, *P* < 0.001) than those in Fortaleza (*r* = 0.236, *P* < 0.001) [35]. Thus, skin color cannot reflect the actual genetic ancestry of individuals. Dr. Beatriz Marcheco had described this eloquently as “*The classic mirror reflects skin color*; *but the DNA mirror reflects our common ancestors*” [33].

**Table 1:** **Comparison between estimates of genetic ancestry and self-reported race in African and European American populations from 1000 genomes project datasets**

**Self-reported race**	**Ancestral population genetic ancestry**			
	**CEU (%)**		**AA (%)**	
	**Mean ± SD**	**Min–max**	**Mean ± SD**	**Min–max**
European ancestry (CEU) (*n* = 87)	0.976 ± 0.022	0.887–0.994	0.024 ± 0.022	0.006–0.113
African ancestry (YRI) (*n* = 88)	0.013 ± 0.009	0.006–0.073	0.987 ± 0.009	0.927–0.994
African American (AA) (*n* = 61)	0.108 ± 0.152	0.006–0.990	0.892 ± 0.152	0.010–0.980

*CEU* European ancestry, *YRI* African ancestry from Nigeria, *AA* African American.

Empirically speaking, ancestry is estimated using ancestry informative markers (AIMs), which are a set of genetic variations for a particular DNA sequence that appear in different frequencies in populations from different regions of the world. The use of AIMs compares an individual’s polymorphisms at these markers with previously analyzed genomic reference sets from people whose ancestral history is fairly well known. AIMs are used to estimate the geographical origins of an individual’s ancestors, typically expressed as proportions of one’s ancestry that comes from different continental regions [36].

Availability of genetic markers that are ancestry-informative and newly developed statistical methods may overcome concerns regarding race/ethnicity categorization [37]. There is evidence that measures of genetic ancestry can improve clinical care for people of mixed race. For example, physicians assessing lung disease can make more accurate diagnoses when they use a reference standard from the patients’ actual genetic ancestry than self-reported race or ethnicity [38]. A large proportion of Native American ancestry is associated with a greater risk of childhood acute lymphoblastic leukemia. Children with more than 10% Native American ancestry need an additional round of chemotherapy to respond to the treatment [39]. Differences in ancestry proportion in admixed population could introduce variation among individuals of the same race and potentially alter genetic association and the therapeutic efficacy of commonly used asthma therapies, such as β2-adrenergic receptor agonists (β-agonists) [40,41]. So far, pharmacogenetic studies of admixed ethnic groups have been limited to small candidate gene association studies. Large consortium-based whole genome sequencing studies are required to provide a reference “genome map” for population without precise matching reference panel including admixed populations for future genetic/genomic and pharmacogenetic studies.

## Genetic markers used to infer ancestry: autosomal SNPs, Y-SNPs, mitochondrial SNPs, and X-SNPs

Although autosomal SNPs are commonly used as genetic markers to infer ancestry or race/ethnicity membership, haploid such as mitochondria, Y-DNA, and X-lined markers are also important to provide separate stories of ancestry of individuals from paternal and maternal sides [42,43]. Therefore, genetic structure created due to autosomal markers could be different from those of lineage markers (often influenced by political, social, and migration history of individuals/populations).

- Autosomal DNA (testing both sexes) markers: autosomal DNA tests utilize DNA from the 22 pairs of autosomal chromosomes. Autosomal DNA is inherited from both parents. Autosomal testing provides percentages of ethnicity using autosomal DNA SNP test (i.e., ancestry informative markers), and it is the most commonly used test to infer ancestry across diploid genome.
- Y-DNA or Y-SNPs (paternal line testing) markers: a haploid Y-DNA is the paternally inherited non-recombining portion of the Y chromosome, and it tests only for males. The Y-DNA testing tests the Y chromosome which is passed intact from father to son with no DNA from the mother. Y-DNA testing can then be used to trace direct paternal line. Y-DNA remains the same in each generation, allowing us to compare surname from different regions to see if we are from the same family. Y-line testing does not indicate anything about the contributions of the other ancestors in a family tree. In other words, you could be 3/4th Native American, with only the direct paternal line being European, and this test would tell you nothing at all about those other three Native lines. When testing the Y-chromosome, there are two types of tests, short tandem repeat (STR) and SNP markers. STR tests are best for recent ancestry while SNP tests tell about more ancient ancestry.
- Mitochondrial DNA (maternal line testing) markers: mitochondrial DNA or mtDNA haploid is the maternally inherited mitochondrial genome (mtDNA) [44]. All children inherit mtDNA from their mother, with no admixture from the father. Like Y-line DNA, mtDNA is passed intact from one generation to the next but through maternal line. Mitochondrial DNA does not follow any surname. In fact, the surname changes in every generation when women marry. Polymorphisms of mtDNA have been used to understand human population distribution around the world. Before modern human traveled across the world, mitochondrial haplogroups were largely restricted to the geographic regions of their origin [45]. For this reason, they are often superimposed on maps of the globe as representative of the human populations derived from those regions of the planet. The mitochondrial genome is a critical target for inherited disparity due to ethnic-based diversity, which is greatest within Africa. Because of the clear associations of mitochondrial haplogroups and ethnic categories with geography, one might naively expect a simple correlation between the two classifications. While, for instance, there is broad correspondence between the L haplogroups and African ethnicity assignments, African ethnicity assignments are present to varying degrees in virtually every haplogroup analyzed and almost every haplogroup contains members of each of the four ethnicities. This is not particularly surprising due to the fact that mitochondrial DNA represents only a very small segment of the complex mosaic of a human’s genetic ancestry, and it suggests that the ability to infer coarse ethnic identity from mitochondrial sequence would be very limited. In fact, studies found that mitochondrial DNA can be used to infer the probable assignment of coarse ethnicity with almost 90% accuracy [46]. This level of accuracy in predicting investigator-assigned ethnicity could be very useful in forensic investigations [47].
- X chromosome (X-DNA testing) markers: an X chromosome DNA test looks at markers on X chromosome(s). Males have one X chromosome that they inherit exclusively from their mother, and females have two X chromosomes that they inherit from both parents, one from their father and one from their mother. This creates a unique inheritance pattern that may provide many insights into one’s maternal heritage. STR markers on the X chromosome have been used in population genetic studies and forensics.

There are two main benefits in using haploid (Y-DNA and mitDNA) markers over diploid (autosomal) markers: 1) they lack recombination. This allows for more easily recoverable phylogenies than is possible for the autosomal markers, allowing for the easier identification of geographically restricted clades, which could be indicative of past historical migration. The second benefit in using the sex-specific systems is their 2) much small effective population size related to autosomal markers due to their haploid mode of inheritance through one sex only. Genetic diversity of present-day American populations is very complex due to the demographic events that resulted in extremely admixed populations [48]. Through the analysis of lineage markers such as mtDNA andY-DNA, it is possible to isolate the original Native American lineages without the confounding effects of admixture due to the absence of recombination. The Native American share was conserved through the maternal line. Since only the egg, not the sperm, contains cytoplasm, we can use this to distinguish the original mother. Studies have shown that the “Eve” for Cuban population is about 38.8% African, 34.5% Native Americans, and 26.7% Europeans. Conversely, by using the Y chromosome, studies have shown that 82% of Cubans are descendants of European fathers, 17% of African fathers, and 1% of indigenous fathers [33,34].

## Multi-locus ancestral haplotype as ancestry-informative regions (AIRs)

Although variation in humans reflect genetic differences at single allele as well as haplotype level, most local ancestry estimators use allele frequency data (locus-by-locus) between parental contributions along the chromosome, ignoring molecular information that is available in haplotype block structure. Individual mutations carry only weak signals about population ancestry. By adding information across the whole genome at haplotype level, we can reconstruct these admixture events more accurately. It has been described that less than 50% of admixture is hard to detect from single locus (or non-recombining genome) data. The power of detecting ancestry switch points between European and African ancestry per person becomes feasible as more and more loci are identified [49]. This approach is referred to as haplotype sharing [50] and involves sharing several markers to identify regions of interest [51] rather than relying on differences in allele frequencies at individual markers. However, previous methods do not take into account multiple loci as provided by haplotype structure in ancestral populations. Potential advantages of multipoint ancestral haplotypes include: (1) their use of more information in the data when a susceptibility variant in the region is untyped or partially typed and (2) the fact that likelihoods at nearby variants are based on the same data, so they are formally comparable for the purposes of localization. As a result, multipoint ancestral haplotype methods have the potential to vastly improve and provide high-resolution localization of variants over single-point methods [52]. By considering the genealogy of ancestral haplotype rather than pairs of variants, this approach may allow the joint estimation of other interesting parameters in the admixture model, such as admixture time, divergence time, population size, and mutation rate as described by Wang [53].

In a founder population, patients with a genetic disease are likely to share predisposing genes from a common ancestor. Depending on the distance of the relationship, patients are expected to share extended segments of DNA around the disease gene, thus the extent of linkage disequilibrium (LD) between the disease and the surrounding marker (about 1 cM) is small enough to be meaningful and large enough to be observed. Because of the size of the shared segment, a genomic search with DNA markers for such regions can efficiently locate the map position of genes using identity by descent (IBD) mapping [50]. IBD mapping is a haplotype sharing statistic (HSS) approach, which uses (hidden) co-ancestry between affected individuals from a founder population. Recently, IBD mapping has been proposed as a useful approach to map genes in a founder population [50]. IBD mapping uses haplotype sharing at several markers rather than differences in allele frequencies at individual markers to identify regions of interest [51]. Devlin et al. [54] described the possibility of mapping disease genes by analyzing excess haplotype sharing. Using this idea, one could integrate information on LD structure of genotype data and interrogating various SNP densities of the current SNP chips, under various disease models and various levels of informativeness among markers between the ancestral populations to better optimize the power of LD admixture mapping procedures and make them more efficient and powerful to identify and localize liability genes for complex diseases including asthma [36].

Limitations related to ancestry markers include the reference sets, which are comprised of the genomes of relatively few sampled individuals who are themselves from a relatively few, geographically restricted regions. Thus, to what extent is a panel derived by contrasting a “Yoruban” sample with “Europeans” appropriate for use in African-American samples? How much is the Yoruban population represents Africa and hence African Americans are debatable [37]. However, the same can be said to the CEU population where recent high-density SNP studies showed population gradient including linkage disequilibrium discrepancies across the North–south and even within Finland (East–west) [55]. Therefore, it is prudent to recognize the limitations of ancestry informative markers in genetic/genomic studies of admixed population.

## Genetic ancestry and clinical predictive variables

Clinical asthma outcome variables such as pulmonary function tests (PFTs) include forced vital capacity (FVC, a measure of lung size), forced expiratory volume in 1 s (FEV1, a standard measure of lung function), and FEV1/FVC ratios. The variation in ancestry in relation to these clinical predictive variables may help to explain differences in disease phenotypes among ethnic subgroups. Recent study showed that in Mexican Americans, European ancestry was associated with more severe asthma, as measured by FEV1, a quantitative measure of lung function. A decrease of 1.7% baseline FEV1 was observed per 10% increase in European ancestry [56]. FEV1 is a measure of airway caliber and a standard measure of lung function, and FEV1/FVC ratio is a commonly used outcome to assess airway obstruction [57]. Age-, race-, and ethnic-appropriate reference equations will be used for PFT results [57-59]. A recent study by the NHBLI-SARP case-only cohort indicated the predictive role of PFT in asthma severity [60,61].

**Figure 3:**
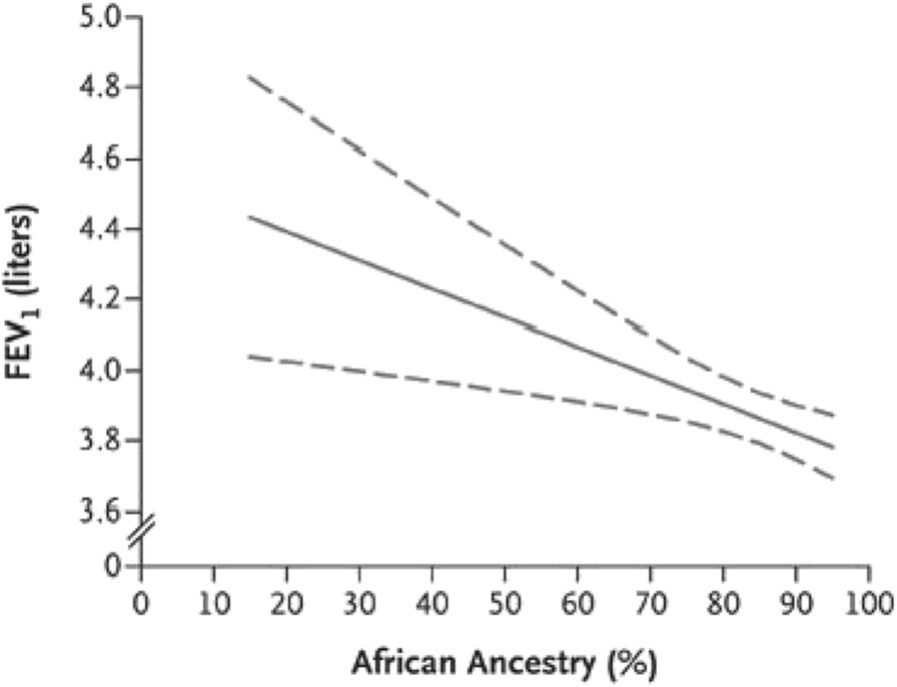
**Relationship of African ancestry proportions with lung function in African-American male subjects using ancestry informative markers.** An inverse relationship between the percentage of global African ancestry and baseline FEV1 (Forced Expiratory Volume, measured in liters) are shown. Reproduced from Kumar et al. [38].

Several studies have associated genetic ancestry with numerous clinical endpoints. African ancestry was inversely related to FEV1 (*p* = 0.007), FVC (*p* = 0.0003), and FEV1/FVC (*p* = 0.035) (Table 2, Figure 3) [38,62]. Higher vs. lower proportion of African ancestry, categorized based on median value, has also been shown to be associated with greater decline in the lung function per pack-year of smoking (−5.7 vs. –4.6 ml FEV1 per pack-year) in contrast to the −3.9 ml FEV1 per pack-year smoked observed among European Americans [63]. Additionally, African Americans with higher proportions of African ancestry have a greater risk of losing lung function while smoking. Studies have shown that each percentage increase in African ancestry was associated with an 8.9-ml decrease in FEV1 (*p* = 0.001) and an 11.8-ml decrease in FVC (*p* = 0.0001). Higher African ancestry was associated with a greater likelihood for an asthma-related physician visit (*p* = 0.004) and greater frequency of urgent or ED visits among asthmatics treated with an inhaled glucocorticoid (*p* = 0.01). In African Americans with more severe asthma, the magnitude of decreased lung function associated with African ancestry was twice that observed in the general population (−8.9 ml vs. −4 ml for FEV1 per percentage African ancestry [38]). These investigators found that adding genetically measured ancestry to the standard lung function prediction equations, rather than relying on self-identified race, reduced misclassification and resulted in the reclassification of asthma severity by 5%. It is important to note that although ancestry is associated with asthma clinical phenotypes, SES and related environmental exposure risk factors were not considered in this study and it is not clear whether race is a confounder for existing socio-environmental differences (i.e., may not be directly causal) between races or independent risk factors (serving as surrogate for genetic differences) for asthma risk. Many factors other than ancestry are influencing the development of asthma. A more careful assessment of the degree of ancestry and asthma in larger cohorts while controlling for environmental exposure and other social determinants of health will further our understanding.

**Table 2:** **Studies considering the relationship between degrees of ancestry proportion and asthma and asthma-related outcomes**

**Study population**	**Specific phenotype**	**Study subjects (** ***n*** **)**	**Ancestry type**	**Markers (** ***n*** **)**	**Main findings**	**Reference**
AA	Lung function	2,169	Structure	Variable	Increasing Af ancestry associated with lower FEV1 and lower FVC	Kumar et al. [38]
AA	Asthma, exacerbation	392	Structure	59	Increasing Af ancestry associated with increasingly severe asthma exacerbation in males but not females	Rumpel et al. [64]
AA	Smoking/lung function interaction	1,281	Structure	1,332	Increasing Af ancestry associated with lower FEV1 per pack-year of smoking	Aldrich et al. [63]
LA	Asthma severity	362	IBGA	44	Increasing NA ancestry associated with less severe asthma	Salari et al. [56]
Puerto Ricans	Lung function	416	LAMP	85,059	Increasing Af ancestry associated with decreased FEV1 and FVC pre- and post-bronchodilator	Brehm et al. [19]

Modified from Goetz et al. [62]. The structure is model-based clustering method; IBGA is a maximum likelihood-based clustering method, and LAMP is a local ancestry in admixed population inference method.

*Af* African, *Am* Amerindian, *As* Asian, *E* European, *NA* Native American, *AA* African American, *LA* Latino American, *FEV1* forced expiratory volume in 1 s, *FVC* forced vital capacity.

## Consortia- and self-reported race/ethnicity information

**Figure 4:**
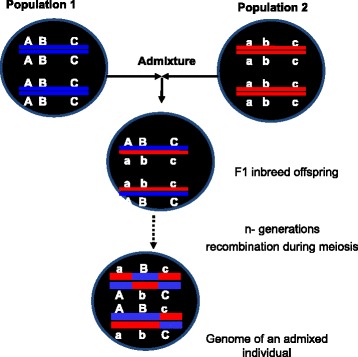
**Schematic representation of genomic mosaicism as a result of ancestral admixture.** An admixed individual derived from two founders in several generations of recombination. The chromosomes of the two founders (shown in different colors) are combined by several generations of random mating to produce present day admixed individual. A DNA sequence of any admixed individual is a mosaic of its founders’ DNA segments. A classic example in humans is the African-American population. The two ancestral populations, European and African ancestry, are represented by dark blue and red chromosomes, respectively. Individuals in the subsequent generation may or may not receive an intact chromosome of their ancestor. As generations continue, mosaics develop for chromosomes 1 and 2 as a result of recombination during meiosis. Chromosomal block sizes are expected to decay with the number of generations of admixture. Only those meiotic crossovers that occur at loci where the paired homologous chromosomes have different ancestries will cause ancestry blocks to decay in size and can be detected using ancestry informative markers (AIMs).

To increase sample sizes and power, several research groups are implementing meta-analysis to combine multiple consortia projects. The recently established Public Population Project in Genomics (P3G) (http://www.p3g.org/secretariat) promotes collaboration between researchers in the field of population genomics to ensure public access to population genomic data. These resources typically include biological samples (i.e., serum, plasma, and DNA) linked to structured clinical databases (i.e., comprehensive electronic medical records (EMR) data) [65,66] in a consortium setting. Considerable data is often collected with each sample such as age, gender, place of residence, state of health, specific diseases, lifestyle (such as how much exercise, smoking, consume alcohol), and socio-economic background. However, most EMRs collect only limited historical information on the ancestry of the donors. Most often, race/ethnicity ancestry data are missing from the EMRs. In some cases, race/ethnicity is assigned by other party such as study coordinator’s visual inspection at time of enrollment and in others; study participants or their guardians are asked to report a single race/ethnicity that they feel best identifies them or their guardian. Hence, there is “missing ancestry” in most EMR resources [13]. As the world populations increasingly do not fall into conventional homogeneous ethnic categories (and becoming highly admixed), the reliability of self-reported race/ethnicity becomes more challenging in the current massive worldwide efforts of integrating multiple consortia projects. In a recent study, Ritchie et al. [67] analyzed 9,483 samples in the Vanderbilt DNA Databank (BioVU) and found missing ancestry information in 9.2% of the records. They showed that most individuals with missing ancestry cluster are in the European American group. However, for individuals with mixed ancestry, such grouping only predicts “major” ancestral clusters but do not reveal the individual’s number of ancestries and/or admixture proportion. In admixed individuals, where each chromosome is likely to be a mosaic of blocks of DNA from ancestral populations, ancestry varies across different loci or different genomic segments (Figure 4). Inferences of admixture proportions by combining information across multiple loci or blocks provide valuable information in estimating and inferring ancestry. This is necessary since grouping obtained using single locus ancestry will vary between loci in an individual. For instance, we may observe the FY*0 (rs2814778) allele at a locus and conclude African ancestry for an individual, but if we observe the MID 575 (rs140864) insertion polymorphism, which is also on the same chromosome as FY*0, then we would have to conclude European ancestry for the same individual at that locus. As a result, samples with missing ancestry could be potentially a source of false positive and false negative results. The availability of millions of genetic markers at unprecedented levels from next-generation sequencing technologies and multi-locus ancestry-based dataset analysis approach provide greater power than ever to assign individuals with missing ancestries with great accuracy [36]. Thus, although a sample in a biobank with no information on race/ethnicity were thought valueless (or remain as a storage facility with limited practical application in disease genetics), it is now possible to have a good idea of the ancestry of a given sample with missing ancestry information and can be biologically categorized for specific studies. It should be noted that EMRs data are uniquely suited for studies that quantify the impact of ancestry in heterogeneous population and play a role in the development of personalized medicine in which treatments will no longer be one-size-fits-all, instead tailored to the molecular and genetic profiles of each patient based on genomic predictors.

## Limitations of self-reported race/ethnicity and genetic ancestry in disease genetics studies

Recent advance in high-resolution genome-wide genotyping allow the inference of genetic or “biogeographical” ancestry using empirical description of individuals and populations [46]. In determining and quantifying genetic background, this technology can augment or supersede the use of proxy methods, such as self-identified race/ethnicity, physical appearance, language-spoken, or geographical origin, to stratify research participants and maximize their relative genetic homogeneity. As described above, the major problem in performing association studies of admixed populations that are assessed solely by self-reported race/ethnicity as a proxy for genetic ancestry is the possibility of spurious association with false-positive or false-negative results. Self-reported and investigator-assigned ethnicity typically relies on the subjective interpretation of a complex combination of both genetic and non-genetic information including behavior, cultural, and societal norms, skin color, and other influences. It is rarely the case that a study participant will report their ethnicity without errors. Self-reported ethnicity errors may occur for various reasons; some people may not be fully aware of their true ethnicity or only know recent ancestry (or their geographic origin) while others may identify with one ethnic group despite their admixed background. The imposition of racial categories on human populations has been one of the most enduring historical forces that shape our life trajectory [68]. To illustrate, in a recent study, 9 of the 1,247 self-reported African Americans were found to have 100% European ancestry [69]. Similarly, some self-identified European Americans have substantial admixture from African ancestry [70]. Both examples illustrate that researchers should be aware of the limitation of self-identified race and ethnic categories as proxies for genetic ancestry [71,72]. Similarly, although ancestry could play a central role in disease etiology, association studies, and variable drug response, it provides less information in identifying societal construct such as health and income disparities. Furthermore, although disease susceptibility loci can differ in frequency across populations, using genetics as the only basis of explaining for health disparities could reinforce racial stereotypes [73]. Moving forward, the potential of both genetics and race/ethnicity to shed light on health disparities must be considered.

Studies showed that extrapolation of genomics data from genetically homogeneous to genetically structured populations could generate large numbers of false positive and false negative results [13]. Population stratification (or structure) is the existence of groups of individuals within a population that have some degree of reproductive isolation from the rest of the population and for which allele frequencies are likely to be different from the population as a whole. Several approaches have been used to adjust population structure in case–control studies. The most commonly used clustering algorithms is *structure* [74]. Using ancestry informative markers, a) local ancestry tracked from each individual can be compared with the genome-wide average ancestry, and b) individuals whose ancestry is not typical of the population under study can then be excluded [2]. To investigate the genetic relationships among ancestral groups, one could also compare patterns of population divergence using Wright’s *F*_ST_ measure [75]. From the *F*_ST_ analysis, one could reliably identify subpopulations within major geographic regions (i.e., Europe, Africa, Asia, and the Native Americans) that exhibit lower or higher pairwise *F*_ST_ (and, therefore, lower or higher genetic similarities). For populations of complicated admixture or unknown origins, a large number of loci with high resolution need to be genotyped, followed by principal component analysis (PCA) to individual-level genetic data. PCA can detect the presence of population mixture and admixture in a sample and thus can be used to determine the axis of variation in different dimensions based on biogeographical ancestry. Adjustment made using PCA approach increases investigator confidence that genetic association findings are not spurious due to stratification. Finally, characterization of culture, socioeconomic status, and environment should be made in disease genetic study, otherwise any or all “racial/ethnic” differences in disease risk factors can erroneously be attributed to presumed population genetic differences. Methods such as mixed model regression could help investigate the genetic and non-genetic risk factors. The failure to account ancestral background can thus prevent proper characterization of the genetic structure of a given study population, leading to inaccurate prediction of outcome as well as incorrect inferences about the evolutionary factors driving patterns of diversity [76].

## Race/ethnicity in biomedical research

There are two major questions to answer before applying race/ethnicity category in biomedical research. First, is race/ethnicity a valid and reliable approach to ascertain individual ancestry? If so, should race be considered by those who study diseases and patient responses to treatment? Second, how do we define (or is it at all possible) race/ethnicity in the context of biomedical research? In general, people self-report their population origin correctly in terms of major population descriptors (such as Caucasian, African-American, Hispanics, Asian, etc.). However, these descriptions are not good indicators of the genetic composition of individuals, since genetic makeup of individuals are highly heterogeneous, and can be captured only with large dimensional genomic data. Genetic ancestry estimation at the individual level is bringing us closer to more personalized or individualized genetic-based medicine [77]. Genomic researchers in medicine should focus on how genetic association results can be used to understand disease process in a way that can inform the clinical care of racial disparities rather than focusing merely on explaining health differences [78].

**Figure 5:**
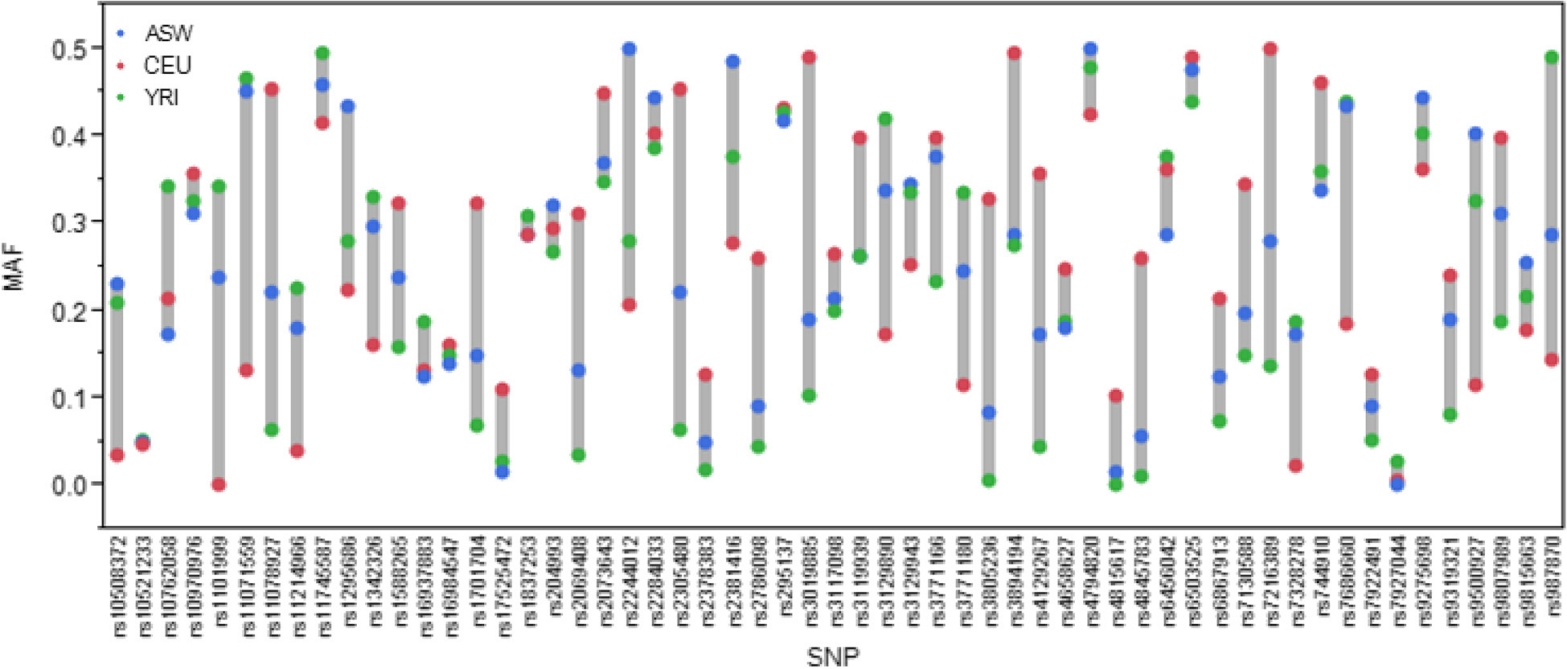
**Minor allele frequency (MAF) distribution.** Asthma-related GWAS SNP’s across African American (ASW), European American (CEU), and African (YRI) populations from the NHGRI GWAS catalog (http://www.genome.gov/gwastudies). The GWAS catalog is an online catalog of SNP trait associations including asthma from published GWAS studies.

Advances in genomic research provide novel insights into individual variation in disease susceptibility and adverse reactions to drugs. However, because of unequal applications of genomics and associated technologies among human populations, the information collected so far does not entirely address disparities at multiple levels. Almost all genetic studies, including many of the identified variants (e.g., asthma) and pharmacogenetic studies have been primarily performed in cohorts of European descent [79]. In European ancestry, genome-wide association study (GWAS) projects that genotype ~1 million tagSNPs in several thousand cases and controls to test for association with disease can capture most of the common variation with minor allele frequencies >5%. However, very dense marker sets must be typed to capture similar variation in African ancestry population. Because of shorter linkage disequilibrium, it has been estimated that a genome-wide association study of an African population would require approximately 1.5 million SNPs to achieve the same resolution as a study of a European population using 0.6 million SNPs [80]. For minority population, few genetic data have been systematically analyzed and the interplay between genetic and various socio-environmental factors remain to be investigated [81,82]. Recent exome study revealed that exomes from individuals of predominantly African ancestry were very different from European ancestry exomes. This is in agreement with the reported genetic diversity between African and European ancestry genomes [83]. Hence, genomic data collection should be extended to as many diverse populations as possible. To illustrate this further, we assess the allele frequency variations at asthma-associated GWAS variants deposited at the NHGRI GWAS catalog (http://www.genome.gov/gwastudies/). Since most GWAS studies are done in populations of European ancestry, we examined the allele frequency patterns of 78 GWAS SNPs associated with asthma and deposited at the GWAS Catalog site. We used 1000 Genomes Project (http://www.1000genomes.org) and AncestrySNPminer (https://research.cchmc.org/mershalab/AncestrySNPminer/login.php) to explore these variants among African American (ASW), African (YRI), and European American (CEU) populations. Although further studies are required to determine the extent to which this variation is responsible for differences in asthma prevalence, the admixed AA population (ASW) exhibited allele frequencies that appear intermediate in relation to the ancestral CEU and YRI populations (Figure 5).

Large consortium-based next-generation sequencing studies such as the NIH/HLBI GO Exome Sequencing Project, the Consortium on Asthma among African ancestry Populations in the Americas (CAAPA), and the 1000 Genomes Project are currently using next-generation whole exome and whole genome sequencing studies to provide diverse genomic information from different admixed populations [84]. These large-scale sequencing projects have revealed that admixed ethnic groups demonstrate a remarkable degree of genetic diversity related to an ancient African ancestry. Such genetic diversity has resulted in shorter regions of shared chromosomal segments (i.e., linkage disequilibrium) and a greater frequency of rare variants in ethnic groups with an African ancestry compared with European ancestral populations. In addition to increased genome and exome sequencing efforts, it is also critical to assess non-genetic factors such as poverty, education, access to health care, cultural practices, and environmental exposure such as traffic, smoke, and mold, which vary substantially among populations and may interact with genetic risk factors.

## Which factors contribute more to health disparity: race/ethnicity or ancestry?

**Figure 6:**
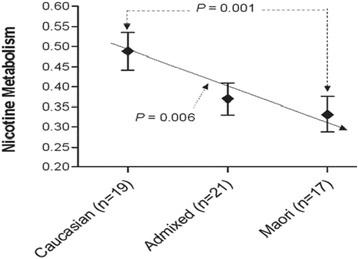
**Nicotine metabolisms in ancestral and admixed population.** Nicotine metabolism was estimated by salivary 3-HC: COT ratio. The *X*-axis shows population groups with sample size in brackets, and the *Y*-axis labels the nicotine metabolism. These data provide evidence that a) Maori smokers have significantly (*p* = 0.001) slower nicotine metabolic rates compared to Caucasian smokers and b) there is a significant linear correlation between nicotine metabolic rate and the degree of Maori ancestry. The admixed population has intermediate nicotine metabolism compared with parental nicotine metabolisms. Reproduced from Lea et al. [93].

Unlike self-reported race-based health disparity studies, which represent a combination of both genetic and environmental background [85], ancestry-based health disparity studies provide a new way to unravel the contribution of genetics to health disparities from non-genetic factors (such as socio-environmental factors). If a greater African ancestry is observed across the genome in asthmatic patients relative to controls, but no significant rise in local ancestry at a particular locus, this may point to a stronger role for socio-environmental factors (e.g., income, education, exposures to traffic, home, cigarettes) independent of ancestry [81,86,87]. Associations found between genetic ancestry and disease could be explained by unmeasured environmental factors that are associated with genetic ancestry and contribute to health disparities, such as socioeconomic status (SES), neighborhood environment, and psychosocial factors including perceived stress or discrimination [88-90]. Therefore, to avoid unwarranted inferences about the magnitude of genetic influences on health disparities, it is critical to include appropriate socio-environmental variables in the analysis of ancestry and disease risk. A good example that illustrates this phenomenon is the recent studies that showed education and socioeconomic factors, but not genetic ancestry, were associated with blood pressure and cancer among African Americans in the US, respectively [91,92]. Furthermore, analysis showed that education was significantly associated with blood pressure in African Americans, but not in European American, suggesting that improved access to education in African American communities may help to reduce racial inequalities in health. An important next step is to explore the mechanisms by which higher education is associated with reduced hypertension and, in particular, why the association is stronger among African Americans than among European Americans. Further studies are also needed to determine whether education is causally related to blood pressure or if it only serves as a marker for other aspects of the social environment. The role of genetic ancestry is also evident from the correlation of nicotine metabolism with admixed ancestry in smokers. Maori smokers on average are slow nicotine metabolizers (~35%) compared to Caucasians (Figure 6). This is mainly because of the significantly higher frequency of slow nicotine-metabolizing variants of the *CYP2A6* gene in Maori compared to that in Caucasians [93]. An admixed individual from Caucasian and Maori showed an intermediate nicotine-metabolism in relation to his or her ancestries. These findings are critical to develop appropriate intervention policies to reduce disease burden due to genetic and non-genetic factors [90].

## Moving beyond race/ethnicity to guide personalized medicine

As the world becomes multiethnic, and intermarriage between different racial/ethnic groups gets more and more common [94], it is increasingly difficult to assign a single ethnicity to an individual. There is a need of clear distinction between personalized medicine and guidelines for the application of personalized medicine in the context of homogeneous and an admixed population. Personalized medicine is a dynamic and broad term used to describe the incorporation of patients’ genomic profiles, family history, and social and other health details into clinical decision-making. Personalized medicine is easier to implement in a more uniform population using the genetic variation present in an individual. In admixed populations, it is much more complex to have a “public health” personalized medicine guideline as the context of the variants may be ancestry-sensitive and on an individual basis. For example, one person may have susceptibility variants that are common in one of their ancestral populations, but not the other (and the other way around for another individual from the same admixed population). In order for the personalized medicine to be meaningful and applicable to the global populations, we will need to know how genetic variants found in different parts of the world influence health and drug response. Thus, the application of personalized medicine should not be limited to patients with well-understood genotypes.

Although knowledge gained in genomics has advanced our understanding of biology, the promise of personalized medicine continues to appear far off for minority and admixed populations. For example, recently, pharmacogenomic information has been added to over 70 drug labels [95], but the studies on which label information are based have mostly focused on European populations. Meanwhile, African populations, who have the greatest genetic variation resulting in more haplotypes, lower levels of linkage disequilibrium, more divergent patterns of linkage disequilibrium, and more complex patterns of population substructure, are grossly underrepresented in the genomic studies that inform pharmaceutical guidance [95]. The result is that clinicians may rely too heavily on data obtained from Europeans to make clinical decisions for Africans and other non-European populations. In addition, this inadequate representation of global populations in the cataloging of genetic variation is hindering the need to move away from the use of group labels such as race, which is often a poor proxy for genetic ancestry. This concern extends to the momentous debate about the development of ‘race-targeted’ drugs, such as BiDil (approved by the US Food and Drug Administration (FDA) to treat heart failure in admixed African Americans), based on subgroup analyses without any adjustment for potential confounders in samples [94]. Intra-ethnic diversity adds complexity to the scientific appraisal, regulatory decisions, and, eventually, prescription of race-targeted drugs. Ignoring admixture or stratification within ethnic groups will complicate the promise of personalized medicine [96-99]. A study by Lee [100] showed that warfarin dosing algorithms that are based on ‘race’ terms for well-defined ethnic groups are not applicable to the heterogeneous admixed population. In April 2011, the American Congress of Obstetricians and Gynecologists (ACOG) adopted a policy to screen all patients for cystic fibrosis because of the difficulty in assigning ethnicity to individuals [101]. The US FDA recommends screening all groups, regardless of race or ethnicity, for the presence of the HLA-B*5701 allele before starting or restarting therapy with Abacavir or Abacavir-containing medications (http://www.fda.gov/Drugs/DrugSafety/ucm123927.htm). Abacavir is used to treat human immunodeficiency virus (HIV) infection. Patients with the HLA-B*5701 allele have a higher risk of developing a hypersensitivity reaction. Furthermore, several medication dosing algorithms around the world are now being developed using the patient’s own genotype data [79,102,103].

## Conclusion

Although conceptual distinction between race/ethnicity and ancestry is widely recognized [104-106], it has not been translated into measurements of how well each accounts for health disparities. Thus, the continued use of race in genetic research obscures the fundamental causes of racial differences in health. Although race and/or ethnicity could serve as good markers to predict socio-economic differentials like housing, income, and/or education, they are poor predictor of genetic ancestry [90]. Increasingly, the world’s populations do not fall into conventional homogeneous ethnic categories, and ancestry informative markers with appropriate statistical methods must be used for quantitative measurement of the genetic ancestry of individuals. Quantifying the contributions of ancestry, environment (such as socio-economic status, life style), and their interactions to disease outcome in the genetically heterogeneous population will be critical to applying genomic-based biomarkers to the practice of medicine. The path to personalized medicine for all ethnic groups requires improvements to our ability to decipher genotype and sequence data using different analysis methods that integrate race/ethnicity information and account for ancestral genetic structure, complex haplotypes, and gene-gene and gene-environment interactions. It is crucial to recognize that disease and health disparities are the products of complex interactions that are not solely limited to genes but also involve environmental factors, socioeconomic status, lifestyle factors, and the biases of health care providers. Thus, it is important to place genetic ancestry factors in context with social, environmental, and economic factors for the purpose of resolving health disparities between populations.

Given higher genetic diversity within races than between races, the use of race/ethnicity as a dissimilarity marker is misleading [107,108]. Genetic ancestry can describe genetic relatedness accurately than race and ethnicity, but it could still exacerbate disparities since it sidesteps the interaction of biological and social factors that contribute to health. The current inference of ancestry based on computer programs with built-in assumptions about how the data should be grouped can sometimes reify racial distinctions by presenting genetic clusters or racial boundaries that do not exist in human population specially in admixed population. In addition, current ancestry inferences are based on reference samples with limited representation of the entire population (e.g., West African ancestry sample for the entire African Americans and Northern European sample for the entire European Americans). Understanding the sources of human genetic variation (using genetic markers) and the causes of health disparities (using race/ethnicity information) could lead to interventions that would improve the public health and bring personalized medicine to all.

## Abbreviations

AIMs: ancestry informative markers
GWAS: genome-wide association study
HapMap: haplotype map of the human genome
LD: linkage disequilibrium
PCA: principal component analysis
SNPs: single nucleotide polymorphism
SVs: structural variants
