# Correction to: Self-reported race/ethnicity in the age of genomic research: its potential impact on understanding health disparities

**DOI:** 10.1186/s40246-021-00322-7

**Published:** 2021-06-15

**Authors:** Tesfaye B. Mersha, Tilahun Abebe

**Affiliations:** 1 Division of Asthma Research, Department of Pediatrics, Cincinnati Children’s Hospital Medical Center, University of Cincinnati, Cincinnati, OH USA; 2 Department of Biology, University of Northern Iowa, Cedar Falls, IA USA

**Correction to: Hum Genomics 9, 1 (2015)**

**https://doi.org/10.1186/s40246-014-0023-x**

Following the publication of the original article [1], an error for reference 69 citation was identified. Reference 69 (Salas A, Carracedo A, Richards M, Macaulay V. Charting the ancestry of African Americans. Am J Hum Genet. 2005;77(4):676–80) referred to a wrong article and was replaced by the following citation:

Kodaman N, Aldrich MC, Smith JR, et al. A small number of candidate gene SNPs reveal continental ancestry in African Americans. Ann Hum Genet. 2013;77(1):56-66.

Also, in the Conclusion section, the word “more” before "accurately" was missing and should be read as: Genetic ancestry can describe genetic relatedness more accurately than race and ethnicity.

The original article [1] has been corrected.
